# Hand-assisted Laparoscopic Colorectal Surgery: Initial Experience of a Single Surgeon

**DOI:** 10.4103/1319-3767.74444

**Published:** 2011

**Authors:** Abdul-Wahed N. Meshikhes, Mokhtar El Tair, Thabit Al Ghazal

**Affiliations:** Department of Surgery, King Fahad Specialist Hospital, Dammam - 31444, Eastern Province, Saudi Arabia

**Keywords:** Hand assisted laparoscopic colectomy, hand port, laparoscopic colectomy

## Abstract

**Background/Aim:**

As totally laparoscopic colorectal surgery is considered challenging and technically demanding with a long steep learning curve, we adopted hand-assisted laparoscopic colorectal surgery as a bridge to totally laparoscopic assisted colorectal surgery. This prospective study aims to highlight the initial experience of a single surgeon with this technique.

**Materials and Methods:**

A prospective analysis of the first 25 cases of hand-assisted laparoscopic colorectal resections which were performed by a single surgeon over a 15-month period. There were 15 males and 10 females with a mean age of 55.5 (range 20-82) years.

**Results:**

The indication in majority of cases was cancer (76%). The procedures consisted of 18 (72%) various colectomies and 7 (28%) anterior resections. The operative time ranged between 110-400 (mean 180) min. There was one conversion (4%) and the mean operative blood loss was 80 (range 60-165) ml. The number of lymph nodes retrieved in the cancer cases was 5-31 (mean 15) nodes. The mean length of hospital stay was five (range 3-10) days. The total number of short-term complications was six (24%) and there was one death due to anastomatic leak and multiorgan failure. Long-term complications after a maximum follow up of 30 months were two incisional hernias at the hand port site, but none of the patients developed adhesive small bowel obstruction or late anastomotic stricture. Currently all our colorectal procedures are conducted laparoscopically.

**Conclusion:**

Hand-assisted laparoscopic colorectal procedures are easy to learn as a good bridge to master totally laparoscopic colorectal surgery.

Since it was first reported in 1991,[1–3] the number of lapraroscopic colectomy (LAPC) procedures remained a minority due to the technical challenges and the long steep learning curve of the new procedure. To overcome such difficulties, a new surgical alternative in the form of hand-assisted laparoscopic colectomy (HALC) was introduced in the mid 1990’s[4–6] This new innovation allows introduction of the surgeon’s non-dominant hand into abdomen through a special hand port while maintaining pneumoperitoneum. A hand inside the abdomen helps to restore the tactile sensation which is lacking in laparoscopic surgery, improves hand-eye coordination and allows safe finger dissection and retraction. This subsequently plays an important role in reducing the operative time. Furthermore, in laparoscopic colonic resection, an incision is needed at the end of the operation to retrieve the resected colon; such an incision may well be utilized at the beginning of the operation. Although this new technique was met by fierce resistance by the laparoscopic community, it is now gaining popularity as adjunct and a bridge towards total laparoscopic colorectal surgery. We report here our initial experience with hand- assisted laparoscopic colorectal surgery which was adopted as a bridge to totally laparoscopic technique, highlighting the short-term and long-term complications.

## MATERIALS AND METHODS

All hand-assisted laparoscopic colorectal procedures performed by a single surgeon were prospectively collected. Patients’ demographic data, indications for the procedure, operative time, length of hospital stay, blood loss, short- and long-term complications and mortality were tabulated. In cases of colorectal cancer, the accurate site of lesion was determined preoperatively by colonoscopy and staging included computerized tomography (CT) scan of the chest, abdomen and pelvis. In case of small lesions that were thought to be impalpable at surgery, endoscopic tattooing of the lesions was performed. All patients were admitted the day before surgery and bowel preparation was routinely started as early as possible to avoid gaseous bloating of the colon during the laparoscopic procedure. Prophylactic antibiotics in the form of a single dose of cefazolin 1 g and metronidazole 500 mg, and DVT-prophylaxis (subcutaneous clexane 40 mg) were administered to all colorectal cases.

### The procedure

**Figure 1:**
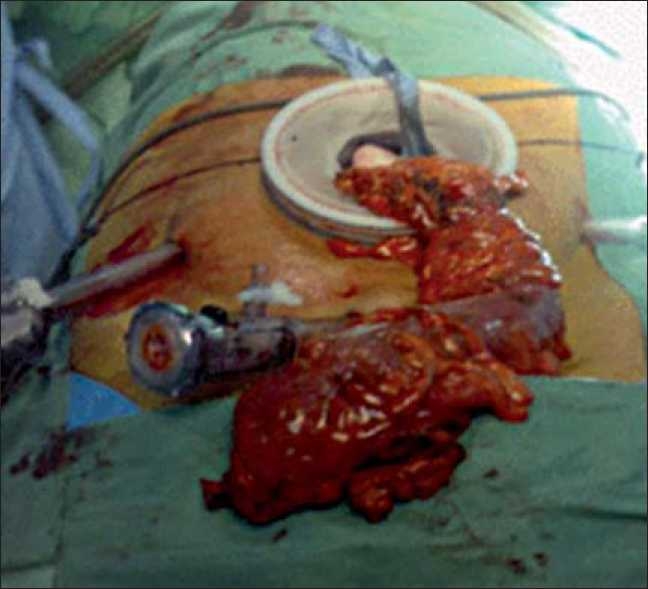
The cancer-bearing colon is exteriorized through the hand port site. The site of trocars’ placement is also shown

At surgery, the patients were placed in supine position and both legs were kept straight and open to allow passage of the circular staplers in left-sided colectomy. The arm and hand of the opposite side of the planned resection are tugged by the patient’s sides. A beanbag is usually strapped to the table and the patient’s shoulders were supported to avoid slippage during the steep reversed Trendelenburg position. The surgeon stands on the left side of the patient for right hemicolectomy, on the right side for left hemicolectomy and anterior resection and between the patient’s legs for transverse colectomy. All procedures were performed through a midline periumbilical incision (6-7 cm long) for placement of the hand port. Another two trocars (10 mm and 12 mm) were also used for the camera and dissection, electrosurgical and stapling devices [Figure 1]. A third 5 mm trocar may be occasionally needed. The dissection starts by medial to lateral dissection after vascular pedicle ligation. This is then followed by lateral dissection. For left hemicolectomy, the colon is transected distal to the lesion at the planned site for the anastomosis and the tumor-bearing segment is exteriorized through the hand port site after application of a wound protector device [Figure 1]. The pathology-bearing colonic segment was then resected extracorporeally and the anvil of a circular stapler is secured with a purse-string around the distal colonic end which is returned into the abdomen. The pneumoperitoneum is then established and the bowel continuity is restored using a circular stapling device through the adequately irrigated rectal stump. All left-sided colonic and rectal anastomoses were carried out intracorporeally, but in all right-sided and transverse colectomies, the anastomoses were done extracorporeally. All left sided anastomoses were tested for proper integrity. A drain was placed selectively whenever it was felt indicated, and wounds were then closed in layers. Postoperatively, all patients are allowed sips of water orally on the evening of the operation, free oral fluids first day, soft diet on the second day, and full diet and discharge home on the third day.

## RESULTS

Over the 15-month period (July 2006- October 2007), 25 patients (15 males and 10 females) with a mean age of 55.5 (range20-82) years underwent hand-assisted laparoscopic colorectal procedures for various indications; the majority (76%) was for cancer. The procedures consisted of seven sigmoid colectomy (one combined with cholecystectomy), seven left hemicolectomy, four right hemicolectomy, and seven anterior resections [Table 1]. All cases were performed electively except one case (4%) who was admitted as an emergency with large bowel obstruction secondary to stenosing cancer of the proximal descending colon. He initially underwent endoscopic stenting, and then was optimized to undergo HALC a week later. The operative time ranged from 110-400 (mean 180) min. There was one conversion (4%) in a patient with fistualizing Crohn’s disease due to dense adhesions and difficulty to identify the right ureter. All rectal cases (*n* = 7) were 8-10 weeks post neoadjuvant chemoradiation. None of the patients needed intraoperative blood transfusion and the mean operative blood loss was 80 (range 60-165) ml. The mean number of lymph nodes retrieved in the cancer cases was 15 (range 5-31) nodes. The mean length of hospital stay was five (range 3-10) days. Since November 2008, all our colorectal procedures have been conducted via totally laparoscopic procedure, while hand-assisted technique is only utilized whenever a difficulty is encountered or conversion is contemplated.

**Table 1:** Indications and the HALC procedures conducted

	Diagnosis	Procedure
Indications for HALC procedures	Cancer: 19 (76%)	
	Colon (*n* = 12)	Right hemicolectomy (*n* = 2)
		Left hemicolectomy (*n* = 6*)
		Sigmoid colectomy (*n* = 4)
	Rectum (*n* = 7)	Anterior resection (*n* = 7**)
	Benign: 6 (24%)	
	Fistulizaing Crohn’s disease (*n* = 2)	Right hemicolectomy (*n* = 2#)
	Diverticular colo-vesical fistula (*n* = 1)	Left hemicolectomy (*n* = 1)
	Sigmoid volvulus (*n* = 3)	Sigmoid colectomy (*n* = 3)

HALC: hand-assisted laparoscopic colorectal procedures,

* One with HAL cholecystectomy also,

** All 8-10 weeks post neoadjuvant chemoradiation,

# One was converted to open due to dense adhesions.

### Complications

Short-term complications: The total number of short-term complications was six (24%); two minor (one wound infections and one prolonged ileus and three major (ischemic stricture, anastomotic leak and iatrogenic left ureteric injury).

The ischemic stricture occurred in a patient who underwent HAL left hemicolectomy for proximal descending colon cancer, T3N1M0. He developed a prolonged postoperative ileus, which settled spontaneously. However, he was readmitted with large bowel obstruction two weeks later necessitating emergency resection of the strictured anastomosis, on-table lavage and a primary end- to-end anastomosis. He subsequently received adjuvant chemotherapy and is doing well at 24 months follow up.

The ileo-colic anastomatic leak occurred on the fifth postoperative day in an octogenarian (the eldest in the series) with some other comorbidities (ischemic heart disease and hypertension). The leak was recognized and intervention was conducted early, but he developed postoperative renal impairment and failure to wean off the ventilator and died six weeks after his second operation. The third was an iatrogenic injury to the left ureter in a male patient with T3N1M0 upper rectal cancer and diverticular disease who was eight weeks post neoadjuvant chemoradiation. Although, the left ureter was clearly identified in its upper part, it was caught accidently in the stapling device during the pelvic transection of the rectum. The injury was recognized immediately and primary end-to-end repair over a double J-stent was carried out successfully.

### Long-term complications

After a maximum follow up period of 30 months, two patients (8 %) developed incisional hernias at the port site requiring mesh repair, but none developed adhesive small bowel obstruction or anastomotic stricture.

## DISCUSSION

This is the initial experience of a single surgeon with hand-assisted laparoscopic colorectal procedures. It is considered as a “learning curve” experience which was utilized as a bridge towards totally laparoscopic colorectal procedures. Prospective randomized and non-randomized trials have confirmed that hand-assisted laparoscopic colectomy (HALC) offers same minimally invasive benefits as laparoscopic colectomy (LAPC). HALC has shorter operative time, lower conversion rate and has comparable complication rate and length of hospital stay.[7–12] However, it has a slightly longer incision size, with increased inflammatory markers e.g. *interleukin 6* and *C-reactive protein* than laparoscopic colorectal procedures.[8] Moreover, HALC patients may need more postoperative medication and have somewhat delayed bowel function and passage of flatus; however, this is of doubtful clinical significance.[13] There is also general agreement that HALC is more suited for the obese and complex colorectal procedures.

HALC was introduced to our institution in July 2006 and was performed solely by one surgeon. The aim was to use this hybrid procedure as a bridge towards totally laparoscopic colorectal surgery. Now after the first 25 cases that constitutes this study group, and since November 2008, all colorectal procedures in the author’s unit are performed via totally laparoscopic-assisted technique. Now, the hand-assisted technique is introduced only in difficult laparoscopic cases or to speed up a certain laparoscopic step such as taking down the splenic flexure and difficult rectal dissection in selected cases. It is also used whenever a conversion to open technique is contemplated.

As our institution is a tertiary oncology center, the majority of our HALC cases were conducted for malignant cases in high number of cases (76%). There was minimal blood loss and none of the patients needed blood transfusion. This can be explained by the ease with which intra-operative bleeding can be controlled by the inserted hand and also due to the use of versatile blood vessel sealing devices such as the harmonic scalpel and Ligasure. It is also noticeable that there were more left sided and rectal than right-sided procedures. This is attributed to the fact that left-sided colonic cancers are more common than the right-sided ones in our area. Furthermore, most right-sided tumors are dealt with by general surgeons in nearby secondary hospitals, while left-sided and especially rectal cases are usually referred to us for management. All cases in our series were done electively except one case who was admitted as an emergency with large bowel obstruction secondary to a stenosing cancer of the proximal descending colon. He initially underwent endoscopic stenting, and then was optimized to undergo HALC a week later. This highlights the emerging trend of endo-laparoscopy for the management of acute left-sided colonic obstruction.

Our initial experience also highlights three interesting short-term complications of laparoscopic colorectal surgery. The first was the ischemic anastomotic stricture, which may be attributed to the stretch on the proximal colon that has occurred during performing the extracorporeal anastomosis. Thrombosis of the terminal vessels feeding the anastomosis was evident during the operation. The second complication was the ileo-colic anastomatic leak in an octogenarian with some other co-morbid conditions. The leak was recognized and intervention was conducted early, but he died of multi-organ failure six weeks after his second operation. The iatrogenic injury to the left ureter was in an elderly man with upper rectal cancer and diverticular disease. The injury was recognized and immediate repair was carried out successfully. It is interesting to note that the ureteric injury occurred in the twenty-fourth hand-assisted case and not early in our experience.

The long-term complication of hand-assisted laparoscopic colectomy has been the center of recent debate. It has been postulated that continuous and persistent stretch of the port site may predispose to the development of incisional hernia. Furthermore, placement of hand in the abdomen in HALC increases the risk of postoperative ileus, development of intrabdominal adhesions with future risk of small bowel obstruction.[14] In our small series and a follow up of less than 36 months, only one case developed postoperative ileus and that was related to an ischemic insult at the anastomosis site. However, two patients developed incisional hernias at the port site which were repaired. A recent report on long-term complications of HALC showed no increased risk of incisional hernia or small bowel obstruction due to adhesions.[14] Compared with the already published literature on HALC, our operative time, blood loss and length of stay were comparable to others [Table 2].

**Table 2:** Comparison of operative time, blood loss, conversion rate, number of complications and mean hospital stay of this series with that previously reported

Author reference (year)	No. of patients	Mean operative time (min)	Mean blood loss (ml)	Conversion rate (%)	Complication	Mean hospital stay (days)
HALS^7^ (2000)	22	152	--	14	1 major	6
Taragarona *et al^8^* (2003)	27	120	--	7	7; 2 major	6
Chang *et al^9^* (2005)	66	189	--	0	14 (21)	5.2
Ringley *et al^10^* (2007)	22	120	75	--	--	4
Chung *et al^11^* (2007)	41	110	35	7	4 (9)	7
Marcello *et al^12^* (2008)	33	175	212	2	2 (6)	5.7
Meshikhes *et al*	25	180	80	4	6; 3 major	5

Figures in parenthesis are in percentage

## CONCLUSION

Hand-assisted laparoscopic colectomy is advocated as a safe and feasible procedure in malignant as well as benign colorectal conditions. It is easy to learn and combine the advantages of both laparoscopic (minimally invasive) and conventional open surgery. It also offers similar short and long-term minimally invasive benefits as laparoscopic colectomy. In our opinion, it is best adopted as an adjunct and a bridge toward adoption of totally laparoscopic colectomy.
